# Intramolecular Charge Transfer of 1-Aminoanthraquinone and Ultrafast Solvation Dynamics of Dimethylsulfoxide

**DOI:** 10.3390/ijms222111926

**Published:** 2021-11-03

**Authors:** Kooknam Jeon, Myungsam Jen, Sebok Lee, Taehyung Jang, Yoonsoo Pang

**Affiliations:** Department of Chemistry, Gwangju Institute of Science and Technology, 123 Cheomdangwagi-ro, Buk-gu, Gwangju 61005, Korea; kooknam@gm.gist.ac.kr (K.J.); watqdt@gist.ac.kr (M.J.); leesebok@gist.ac.kr (S.L.); taehyungjang@gist.ac.kr (T.J.)

**Keywords:** intramolecular charge transfer, excited-state dynamics, solvation dynamics, coherent oscillations, femtosecond stimulated Raman spectroscopy

## Abstract

The intramolecular charge transfer (ICT) of 1-aminoanthraquinone (AAQ) in the excited state strongly depends on its solvent properties, and the twisted geometry of its amino group has been recommended for the twisted ICT (TICT) state by recent theoretical works. We report the transient Raman spectra of AAQ in a dimethylsulfoxide (DMSO) solution by femtosecond stimulated Raman spectroscopy to provide clear experimental evidence for the TICT state of AAQ. The ultrafast (~110 fs) TICT dynamics of AAQ were observed from the major vibrational modes of AAQ including the ν_C-N_ + δ_CH_ and ν_C=O_ modes. The coherent oscillations in the vibrational bands of AAQ strongly coupled to the nuclear coordinate for the TICT process have been observed, which showed its anharmonic coupling to the low frequency out of the plane deformation modes. The vibrational mode of solvent DMSO, ν_S=O_ showed a decrease in intensity, especially in the hydrogen-bonded species of DMSO, which clearly shows that the solvation dynamics of DMSO, including hydrogen bonding, are crucial to understanding the reaction dynamics of AAQ with the ultrafast structural changes accompanying the TICT.

## 1. Introduction

Charge transfer has been considered as one of the fundamental photoinduced processes in many chemical and biological systems including oxidation and reduction reactions and natural photosynthesis [1,2,3,4,5,6]. Intramolecular charge transfer (ICT), as one of the model systems for charge transfer, has been extensively studied experimentally and theoretically, whereby the electron donor and acceptor groups are connected by a single chemical bond or π-bridge [7,8,9]. Since the ultrafast charge transfer processes are often observed in natural photosynthesis with the utmost quantum efficiency, optimal molecular structures for efficient ICT processes have been of great importance for the dyes and sensitizers adopted in various applications including for organic light-emitting devices, photovoltaics, solar cells, etc. [1,10,11,12,13]. Ultrafast structural evolutions of chromophores during the ICT processes, such as the *twisted* ICT (TICT) or *planar* ICT (PICT) state in the excited states have been investigated by many time-resolved electronic or vibrational spectroscopy and time-dependent density functional theory (TDDFT) simulations [9,14,15,16,17].

Furthermore, 1-aminoanthraquinone (AAQ) is one of the model systems for the ICT, where the amino group attached to the backbone of anthraquinone can be twisted during the ICT process in the excited states from the planar geometry in the ground state. The steady-state absorption and emission measurements of AAQ showed that the fluorescence of AAQ is strongly dependent on the solvent polarity and large Stokes shifts are observed in polar solvents [18,19,20]. Theoretical investigations of AAQ through TDDFT simulations suggested that the ICT process occurs with a relatively low energy barrier (Δ*E*_a_ ≈ 350 cm^−1^) in acetonitrile with a twist of the amino group [21]. Additionally, the intramolecular proton transfer reaction of AAQ is estimated as implausible in the S_1_ excited state due to the high energy barrier (Δ*E*_a_ ≈ 3300 cm^−1^) of the proton transfer [22]. In recent transient absorption measurements of AAQ in ethanol, an excitation-dependent TICT dynamics of 75–240 fs and a slower PICT dynamics of 4.1 ps have been separately measured. The decay of the PICT emission was observed with the intersystem crossing to the triplet state [23,24]. However, no clear experimental evidence for the structural changes of AAQ such as the twist of the NH_2_ group in the excited state has been presented.

The nuclear coordinates of the chemical reaction must be strongly coupled to other vibrational modes of the reactant, which is part of the complicated nature of the potential energy surface of polyatomic molecules. Ultrafast electronic or vibrational spectroscopy and the nuclear wavepacket analysis often evidence the coherent oscillations in the electronic or vibrational transitions in the excited state, originating from the couplings between the nuclear coordinates [25,26,27,28,29,30,31,32,33,34,35,36]. Mathies and co-workers reported various quantum beatings in the time-resolved Raman spectra of chromophores within the protein environment, where the restricted vibrational motions such as the wagging vibrations in a protein pocket were directly related to the coherent oscillation [26,37,38,39,40,41,42]. The coherent signals, originating from the coupling of potential energy surfaces, carry crucial information about the reaction coordinate and the excited-state dynamics. In chemical reactions occurring in the solution phase, the reaction dynamics of reactants are inherently dependent on the solvent properties. Effective solvation may facilitate chemical reactions, and vibrational cooling can stabilize the product state by dissipating the excess vibrational energy after chemical reaction [43,44]. Coherent nuclear wavepacket motions, resulting from the strong solvation via hydrogen bonding, have been reported for chemical reactions in the solution phase [45,46,47,48].

In this paper, the excited-state dynamics of AAQ were studied using time-resolved electronic and vibrational spectroscopy. The ultrafast ICT process involving the twist of the amino group was found to be strongly coupled to other solute vibrational modes and to solvent vibrational modes of dimethylsulfoxide (DMSO). We report that the excited-state dynamics investigation by FSRS may provide important experimental results for the understanding of chemical reaction dynamics in the liquid phase.

## 2. Results and Discussion

**Figure 1:**
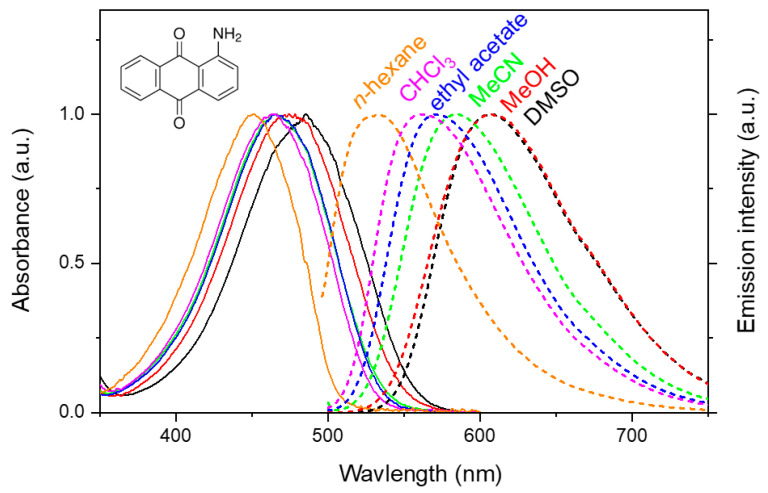
Absorption and emission spectra of the 1-aminoanthraquinone (AAQ) in a number of solvents. The excitation at 485 nm was used for the emission measurements and all the spectra were normalized for comparison.

The steady-state absorption and emission spectra of AAQ in several solvents are presented in Figure 1. The absorption and emission bands of AAQ both show strong solvatochromic shifts. The absorption band, appearing at 451 nm in nonpolar *n*-hexane, shifts to longer wavelengths as the solvent polarity increases. The absorption maximum of AAQ appears at 485 nm in a polar DMSO solution. The emission band of AAQ, centered at 532 nm in *n*-hexane, shows similar but much larger red-shifts with the increase of the solvent polarity, up to 607 nm in a DMSO or methanol solution. The Stokes shift of AAQ in a polar DMSO solution (4140 cm^−1^) is larger than that in the *n*-hexane solution (3380 cm^−1^), which may represent the charge transfer character of the excited states of AAQ. The dipole moment of AAQ increases in the excited state by 1.5–6.3 D, as estimated from the solvatochromic methods [49]. The quantum yields of AAQ decrease with an increase of the solvent polarity (0.15 in *n*-hexane and <0.03 in DMSO) [50]. The increases of the Stokes shifts and the dipole moment in the excited state, and the decreases of the quantum yields in polar solvents all support the existence of the ICT state in the excited state of AAQ.

**Figure 2:**
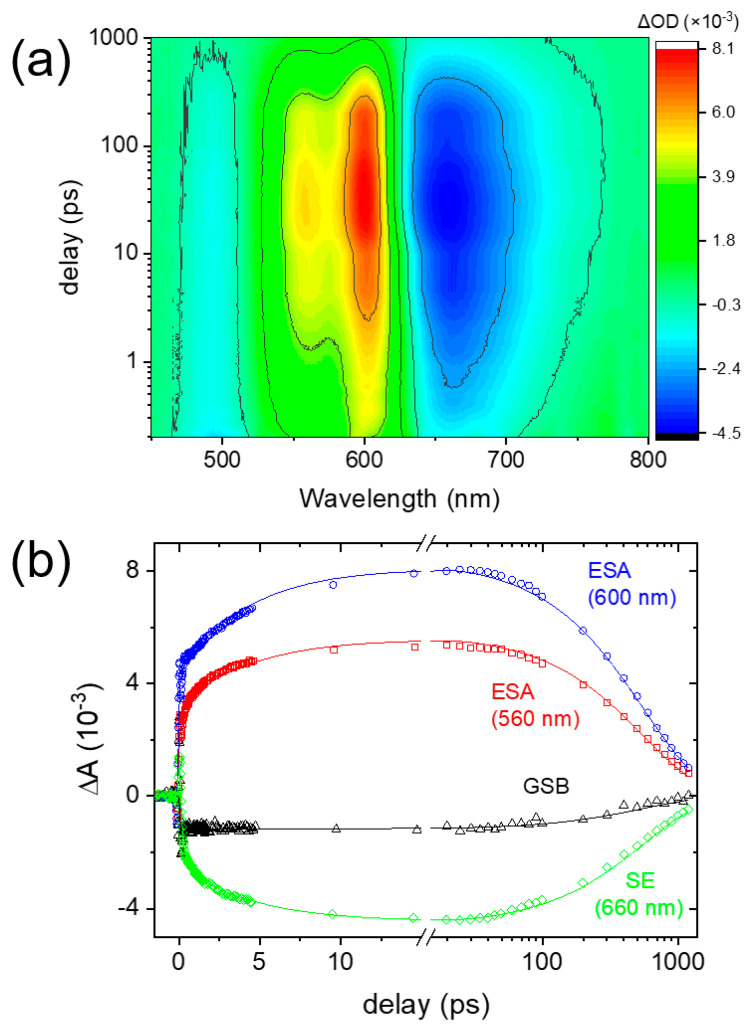
Transient absorption results of 1-aminoanthraquinone (AAQ) in DMSO with 403 nm excitation; (**a**) false color map and (**b**) kinetics for the ground state bleaching (GSB) at 485 nm, excited-state absorption (ESA) at 560 and 600 nm, and stimulated emission (SE) at 660 nm with the Gaussian-exponential fit lines.

The transient absorption spectra and the kinetics of AAQ in DMSO obtained using 403 nm excitation are shown in Figure 2. The positive bands at 560 and 600 nm are the excited-state absorption (ESA) and a broad negative band centered at 660 nm represents the stimulated emission (SE) of the S_1_ state. The positive ESA bands at 600 nm seem to subtract the low-wavelength side of the SE bands substantially. Thus, the SE bands appear strongly red-shifted from the steady-state emission band (centered at 607 nm). Therefore, the SE bands at 660 nm represent the emission of AAQ from the ICT state. The negative band, which is centered at 490 nm, represents the ground state bleaching (GSB). The kinetics of the ESA, SE, and GSB bands shown in Figure 2b were fit with three exponential functions that were convoluted with the Gaussian instrument response function in a global analysis [51,52], where three kinetic components of 180 fs, 4.7 ps, and 560 ps were obtained. The ESA and SE bands showed a biexponential increase of 180 fs and 4.7 ps, revealing that these dynamics should be related to the ICT state. The 560 ps component represents the fluorescence lifetime which is similarly observed from the decay of GSB. The 4.7 ps component is considered as the vibrational relaxation dynamics in the ICT state, which is often observed for small organic chromophores in the excited-state potential surface [48,53,54,55,56]. Then, the fastest 180 fs component can be assigned as the ICT dynamics of AAQ in the S_1_ state. However, the ESA or SE band of AAQ in the LE state, representing the Franck–Condon region, reached by photoexcitation from the ground state, was not separately observed, which is possibly due to the small oscillator strengths between the upper excited and ground states. Moreover, the structural changes of AAQ accompanying these excited-state dynamics are not quite clear between the TICT and PICT states.

Recently, the transient absorption results of AAQ in ethanol have been reported [23,24]. The excited-state dynamics of AAQ in ethanol appeared strongly dependent on the excitation wavelength. The ultrafast ICT dynamics of 75–240 fs were observed with the excitation pulses which placed excess vibrational energy in the S_1_ excited state [24], similar to the 180 fs kinetics observed in the DMSO solution. We also measured the transient absorption of AAQ in DMSO with the 500 nm excitation, where the ultrafast ICT dynamics of 150 fs were also observed (see Figure S1 in the Supplementary Materials). Since the excited-state dynamics of AAQ in DMSO are not dependent on the excitation energy, the excited-state intramolecular proton transfer (ESIPT) across a high energy barrier in the S_1_ excited state is not considered plausible [24]. In the previous transient absorption measurements of AAQ in ethanol, the intersystem crossing with a 550 ps time constant to the triplet state was observed, but the phosphorescence band centered at 770 nm was not observed [21,23,24]. As shown in Figure 2b, the recovery of the GSB band is almost completed in a 1.2 ns delay time and the transient absorption signals of the triplet states were not observed. Thus, the intersystem crossing to the triplet state is considered much less significant in a DMSO solution compared to the case of an ethanol solution. However, the assignment of the ICT dynamics of 150–180 fs requires further experimental evidence of the structural changes of AAQ accompanying the ICT, although a twist of the amino group has been suggested by the theoretical investigations based on TDDFT simulations [21,23,24].

**Figure 3:**
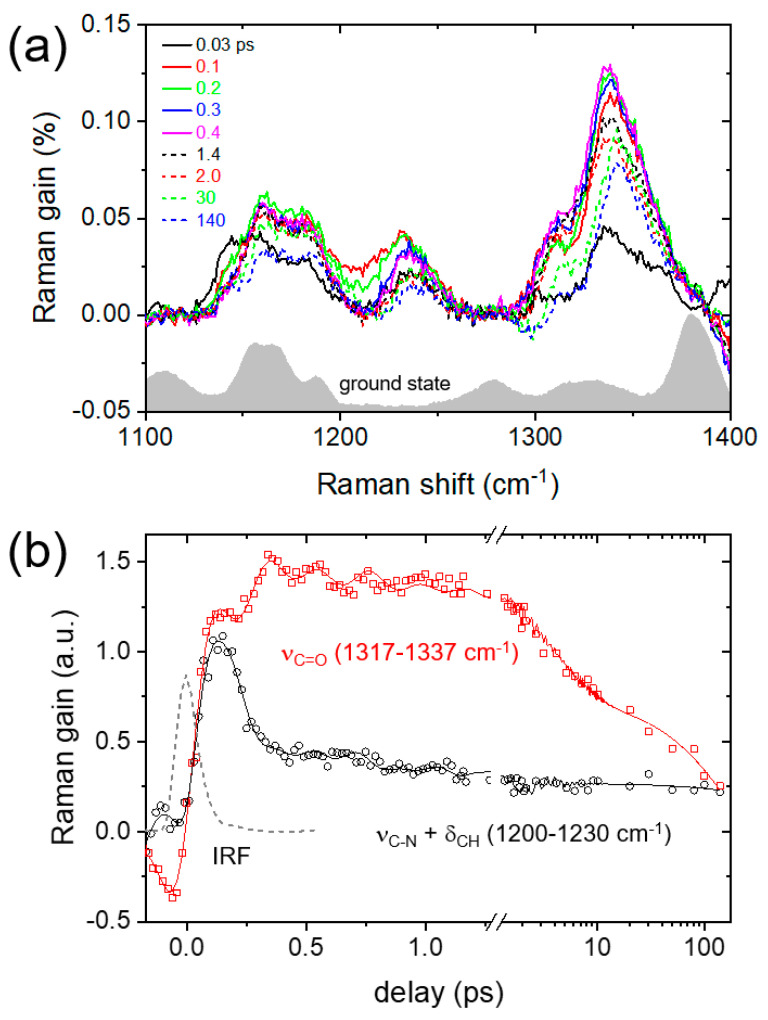
(**a**) FSRS of AAQ in DMSO with 403 nm excitation were compared with the ground-state spectrum; (**b**) the kinetic traces of the ν_C-N_ + δ_CH_ (1200–1230 cm^−1^) and ν_C=O_ (1317–1337 cm^−1^) modes. The fit results with Gaussian-convoluted exponential and damped oscillator functions were shown as solid lines and the instrument response function (IRF) of the FSRS measurements obtained from the ν_S=O_ of DMSO was also displayed.

The FSRS of AAQ in DMSO which was obtained with a 403 nm excitation are shown in Figure 3. All the transient Raman spectra are shown as difference spectra from the ground-state spectrum of AAQ taken at a negative time delay (−5 ps). Several Raman bands of AAQ at 1145, 1162, 1236, 1308, and 1340 cm^−1^ appeared in the excited-state spectra, which is distinct from the ground-state spectrum. The additional skeletal vibrational bands at 1527 and 1590 cm^−1^ are also shown in Figure S4 in the Supplementary Materials. The excited state Raman spectrum of AAQ displays ultrafast changes in many vibrational modes. The Raman spectra at a 0.0–0.1 ps delay with the δ_NH2,__rocking_ at 1145 and ν_C-N_ + δ_CH_ broadly at 1200–1230 cm^−1^ are different from those at a 0.4 ps or later delays with several vibrational modes, δ_CH_ at 1162, ν_C-N_ + δ_CH_ at 1236, ν_ring_ at 1308, and ν_C=O_ at 1337–1340 cm^−1^. No apparent vibrational changes related to the ESIPT of AAQ in the excited state, for example, the appearance of ν_C=N_ and the disappearance of ν_C=O_ were observed. Thus, all the spectral changes of AAQ are interpreted as the ICT in the excited state rather than the ESIPT. The vibrational assignments of the excited-state vibrations of AAQ were mostly based on the TDDFT simulations and the previous reports on dihydroxyanthraquinone (see Figure S7 in the Supplementary Materials for details) [48,56].

Figure 3b compares the Raman intensities between the frequency ranges of 1200–1230 and 1317–1337 cm^−1^ which include the population dynamics of the ν_C-N_ + δ_CH_ and ν_C=O_ modes, respectively. It is clear that the Raman intensity of the ν_C-N_ + δ_CH_ mode sharply decreases in 200–300 fs after the initial photoexcitation while the intensity of ν_C=O_ mode increases by a similar time constant as the decay of the ν_C-N_ + δ_CH_ mode. To separate the population dynamics of these modes from the coherent oscillation signals, a sum of Gaussian-convoluted exponential functions and the damped oscillator functions with a couple of sinusoidal functions were used in the fit analysis (see Equation (S2) in the Supplementary Materials) [48,57]. In the global analysis of both vibrational modes, two time constants of 110 fs and 3.1 ps were obtained for the exponential functions in addition to the ~200 ps population dynamics, which is assigned as the time constants for the TICT state formation and the vibrational relaxation in the TICT potential surface, respectively. Similarly, 110 fs dynamics was also observed for the ultrafast decay of the δ_NH2, rocking_ at 1145 cm^−1^, and the vibrational relaxation dynamics of 3 ps is further confirmed by the population dynamics of many ICT bands including the bands at 1236, 1340, 1527, and 1590 cm^−1^ (see Figure S4 in the Supplementary Materials). Furthermore, the blue-shifts of 3–5 cm^−1^ that are commonly observed in these vibrational modes show a similar 3 ps vibrational relaxation dynamics in the TICT state, which is generally considered as evidence for the vibrational relaxation in the anharmonic potential surface [55,58]. The differences between the vibrational relaxation dynamics obtained from FSRS and the transient absorption measurements are then considered as the difference between the TICT and the PICT dynamics of AAQ in the S_1_ excited state. While transient absorption observes the overall PICT dynamics of AAQ (150–180 fs), and the subsequent vibrational relaxation of the PICT state (4.7 ps), the FSRS results are more sensitive to the TICT dynamics (110 fs) and the vibrational relaxation in the TICT state (3.1 ps).

The FSRS of AAQ in DMSO exhibited clear evidence for the ultrafast (110 fs) internal conversion to the TICT state. The δ_NH2,_ _rocking_ at 1145 and ν_C-N_ + δ_CH_ at ~1215 cm^−1^ would represent the vibrational bands of the LE state and several vibrational bands including the ν_ring_ at 1308 and ν_C=O_ at 1340 cm^−1^ would represent the TICT state in the S_1_ potential surface. Since the major vibrational changes with the TICT were observed in the vibrational modes of the amino (-NH_2_) group and the adjacent carbonyl group, the structural changes of AAQ such as the twist of the amino group seem quite reasonable with our FSRS results.

**Figure 4:**
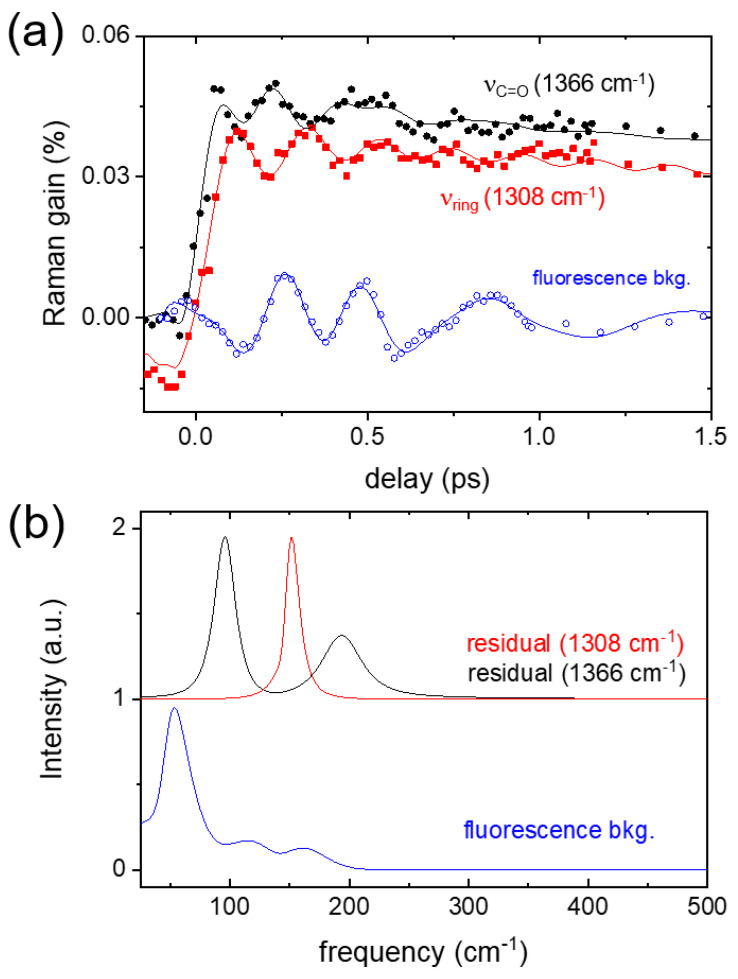
(**a**) The kinetic traces for the vibrational intensities centered at 1308 and 1366 cm^−1^ in the FSRS of AAQ in DMSO with 403 nm excitation; the Gaussian-convoluted exponential fit results were shown in solid lines and the coherent oscillation signals in the fluorescence background were also displayed; (**b**) fast Fourier transformation (FFT) results of the residuals from the Gaussian-convoluted exponential fit results of the 1308 and 1366 cm^−1^ modes, and the coherent oscillation signals in the fluorescence background.

It is interesting to note that many excited-state vibrational bands include the coherent oscillation signals in the Raman intensity up to the time delay of ~1 ps. Figure 4a compares two coherent oscillation signals contained in the ν_ring_ and ν_C=O_ modes of AAQ probed in a 5 cm^−1^ bandwidth centered at 1308 at 1366 cm^−1^, respectively. The vibrational intensities in these frequency ranges appear insensitive to the ultrafast TICT process (110 fs) which is presented in Figure 3b. Instead, the out-of-phase (~180°) oscillation patterns with a common frequency of (~250 fs)^−1^ were resolved. The coherent oscillation signals contained in both vibrational bands were retrieved from the kinetic fit with the sum of the Gaussian-convoluted exponentials and the damped oscillator functions (see Equation (S2) in the Supplementary Materials). The transient absorption signals of the sample were mostly removed via pump-probe type measurements by the Raman pump, but small amounts of the stimulated emission spectrum were still observed as the background signals in the FSRS measurements. The fluorescence background signals that are displayed together in Figure 4a were obtained at the Raman shift of 560 cm^−1^, where no apparent vibrational mode of the ground or excited state exists nearby. Interestingly, the fluorescence background signals also show the coherent oscillations with a slightly different frequency compared to (~250 fs)^−1^ in the oscillation signals of AAQ vibrational modes at 1308 and 1366 cm^−1^.

The coherent oscillations in the vibrational intensities of the 1308 and 1366 cm^−1^ bands and the fluorescence background signals, were obtained using the linear prediction singular value decomposition method [59,60]. As shown in Figure 4b, the fast Fourier transformation (FFT) spectra for the coherent oscillation signals in the ν_ring_ and ν_C=O_ modes of AAQ showed the major spectral components at 96, 152, and 194 cm^−1^ while the results from the fluorescence background signals showed a major peak at 54 cm^−1^. Although limited spectral information on the strongly coupled vibrational modes to the reaction coordinate of the TICT was obtained with the FSRS measurements, it is clear that the anharmonic couplings of the TICT reaction coordinate of AAQ are different from those strongly coupled to the reaction coordinate of the PICT, leading to the emitting PICT state of AAQ.

The coherent oscillations in the ν_ring_ and ν_C=O_ modes of AAQ during the TICT may originate from modulation with the low-frequency vibrations of the amino and the adjacent carbonyl groups which may weaken the intramolecular hydrogen bond between the carbonyl and amino groups. The anharmonic coupling of high-frequency vibrational modes to low-frequency vibrational modes of intramolecular and intermolecular hydrogen bonding has been repeatedly observed in numerous time-resolved electronic or vibrational spectroscopy measurements [32,36,61,62,63,64]. Mathies and co-workers reported vibrational coherence in the CH wagging vibrational modes of rhodopsin, which are strongly coupled to the photoinduced *cis*→*trans* isomerization of the retinal chromophore [40,65]. It is interesting to note that the wagging vibrations of the ethylenic CH may facilitate the isomerization of the retinal backbone. Both in-phase and out-of-phase coherent oscillations in the coupled vibrational modes to the reaction coordinate have also been observed in the excited-state proton transfer of green fluorescent protein [26]. From the oscillations in the phenolic C–O stretching and C=N stretching vibrations, the phenol ring wagging vibration of 120 cm^−1^ was suggested for the strong coupling to the reaction coordinate of the proton transfer reaction. Out-of-phase couplings between the ν_ring_ and ν_C=O_ modes of AAQ may also indicate that the conjugated π backbone of anthraquinone is coupled to the rotation of the amino group in the S_1_ state. The transition state of the ICT process of AAQ may induce the electron density changes, which perturb the conjugated π backbone. Furthermore, similar out-of-phase couplings have been reported in the excited-state proton transfer of 1,2-dihydroxyanthraquinone and green fluorescent protein [26,56].

In the TDDFT simulations on AAQ in the S_1_ state with the planar or twisted amino group, several low-frequency vibrational modes which may represent the anharmonic coupling to the ν_ring_ and ν_C=O_ modes of AAQ during the TICT were found. As shown in Figure S8 in the Supplementary Materials, several out-of-plane deformation modes including the 172 cm^−1^ band of the planar conformer and the 183 cm^−1^ band of the twisted conformer of the AAQ, exhibit strong deformations in the amino and adjacent carbonyl groups such as the twist of amino group.

**Figure 5:**
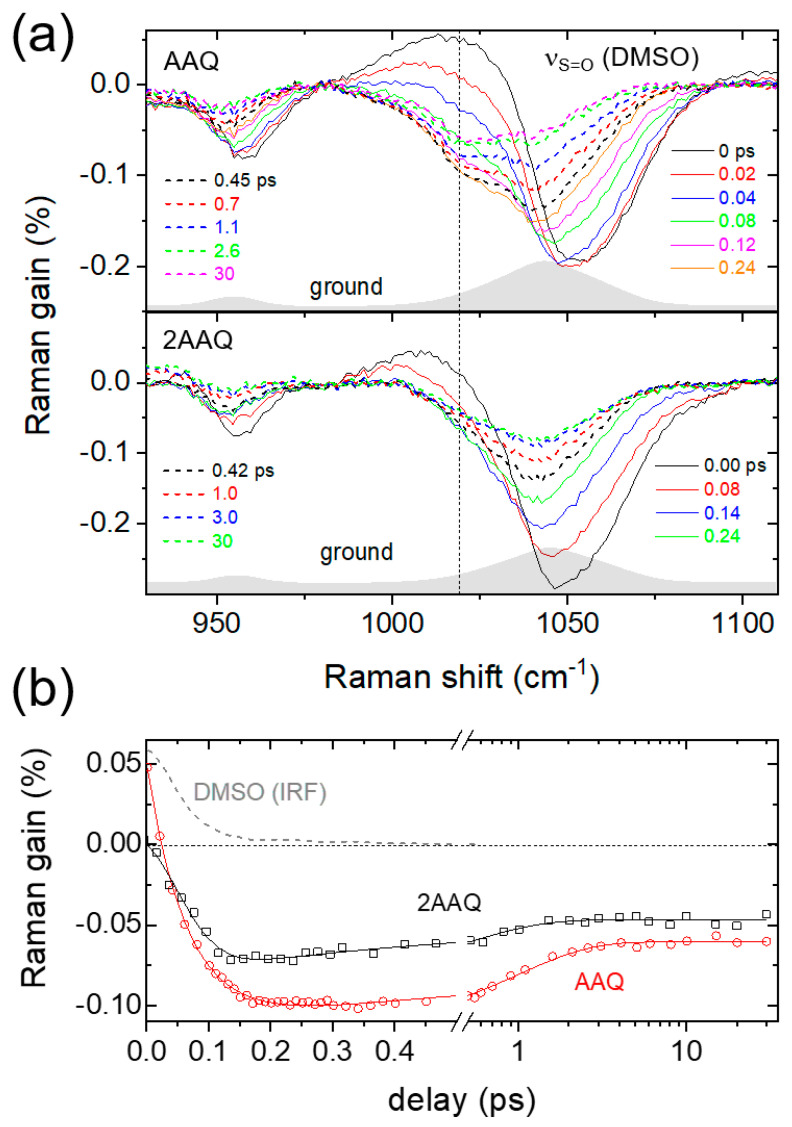
The solvation dynamics of DMSO in the FSRS measurements of 1-aminoanthraquinone (AAQ) and 2-aminoanthraquinone (2AAQ) with 403 nm excitation; (**a**) time-resolved Raman spectra of the ν_S=O_ mode of DMSO, (**b**) the kinetic traces of the hydrogen-bonded sub-band of ν_S=O_ centered at 1020 cm^−1^. The Gaussian-convoluted exponential fit results were shown in solid lines and the instrument response function (IRF) of the experiment obtained only with DMSO at the same frequency was also compared.

The solvent vibrational bands of DMSO also exhibit temporal changes upon the photoexcitation and instantaneous structural changes of solute molecules [48,56]. Figure 5a shows the transient changes in the ν_S=O_ of DMSO obtained in the FSRS measurements of AAQ and 2-aminoanthraquinone (2AAQ; see Figure S10 in the Supplementary Materials for the molecular structure) with a 403 nm excitation. The amino group in 2AAQ is located further apart from the carbonyl group than in AAQ, so the intramolecular hydrogen bonding between these groups is considered absent. At around a zero time delay, the cross-phase modulation (CPM) artifacts appear as dispersive nonlinear signals with a positive peak at 1023 and a negative peak at 1050 cm^−1^ (see Figure S9 in the Supplementary Materials also for the results of DMSO only), which only lasts during the pulse [66]. It is interesting to note that the ν_S=O_ sub-band of DMSO centered at 1020 cm^−1^ shows a sharp decrease in 150 fs and recovers slowly in 2–3 ps with AAQ, which is distinct from those of 2AAQ and DMSO as compared in Figure 5b. The Gaussian-convoluted exponential fit achieves a rapid rise of 60 fs from the ν_S=O_ sub-band at 1020 cm^−1^ with AAQ, which does not occur in the case of 2AAQ or DMSO only. The small negative intensities of 2AAQ around 1020 cm^−1^ at time delays of 0.1–0.3 ps can be still considered as originating from the orientational changes of DMSO molecules in the solvation shellsas the ground state ν_S=O_ modes are centered at 1040 cm^−1^ [48]. However, the presence and absence of the ultrafast (60 fs) negative spectral features centered at 1020 cm^−1^ between AAQ and 2AAQ exhibit the differences in the solvation dynamics of DMSO upon the ultrafast ICT or excited-state dynamics of both aminoanthraquinone isomers. The recovery of the negative ν_S=O_ sub-band intensities centered at 1020 cm^−1^ were also different between the AAQ and 2AAQ. While the ν_S=O_ of DMSO with 2AAQ showed a more rapid 0.5 ps decay, the recovery of AAQ appeared slower with the time constant of 1.0 ps. The recovery of the ν_S=O_ intensities of DMSO with AAQ and 2AAQ are related to the vibrational relaxation dynamics of solute molecules, where the slower solvation dynamics of 1.0 ps observed for AAQ is considered strongly related to the structural changes of AAQ upon the TICT in the excited state. As previously reported with hydroxyanthraquinones in DMSO, the long-lasting negative features in the ν_S=O_ of DMSO with AAQ and 2AAQ in the time delays of 10 ps or later are considered as the vibrationally hot Stokes Raman bands of DMSO [48].

We confirmed that the ultrafast decrease and recovery in the ν_S=O_ of DMSO at 1020 cm^−1^ with AAQ are both strongly related to TICT and accompanying structural changes of AAQ such as the twist of the amino group in the excited state. Baiz and co-workers reported that the ν_S=O_ band of DMSO consists of sub-bands representing the “free” (monomer), “aggregated” (dimer), and “hydrogen-bonded” species based on the infrared absorption measurements and molecular dynamics simulations [67]. The ν_S=O_ bands of “hydrogen-bonded” species of DMSO appear at 1023 and 1012 cm^−1^ in the infrared absorption spectrum depending on the degree of hydrogen bonding. Thus, the decrease in the ν_S=O_ band of DMSO at 1020 cm^−1^ with AAQ represents the decrease of the single hydrogen-bonded species of DMSO, and the solvation dynamics in the ν_S=O_ with AAQ represented by a rapid (60 fs) decrease and slower (1.0 ps) recovery are interpreted as the breakage and reformation of the hydrogen-bonded species of DMSO, respectively, during and after the ultrafast TICT of the solute AAQ.

The ultrafast hydrogen bond cleavage of coumarin 102 in phenol and ethanol upon the dipole excitation of coumarin has been reported using time-resolved infrared and Raman spectroscopy, where the breaking and reformation of hydrogen bonds are indirectly observed from the hydrogen bond-sensitive ν_C=O_ mode of the solute coumarin [68,69,70]. The hydrogen bond cleavage in ethanol occurs with a time constant of ~140 fs and appears faster than the ~200 fs time resolution of time-resolved infrared absorption spectroscopy in phenol. The reorientation of phenol molecules with a time constant of 800 fs has also been observed from the ν_C=O_ of coumarin. In this study, we have similarly measured the instantaneous (60 fs) hydrogen bond cleavages between AAQ and DMSO with the ultrafast TICT of the solute AAQ. The reformation of the hydrogen bond between AAQ and DMSO, possibly due to the reorientation of DMSO molecules in the solvation shells, appears to occur with a time constant of 1.0 ps. It is interesting to note that the solvation dynamics of DMSO observed with the TICT of AAQ in the excited state are distinct from those observed with 1,2-dihydroxyanthraquinone where an instantaneous (100–120 fs) increase and a slower (0.4–0.5 ps) decay in the “free” or “aggregated” species of DMSO were both observed with the ESIPT of the solute, occurring with a time constant of 110 fs [48]. Overall, the ultrafast solvation dynamics of DMSO in the ν_S=O_ or ν_CSC_ modes have been observed along with the ultrafast structural changes of the solutes in the excited state during the intramolecular charge and proton transfers.

Sun and co-workers reported an extensive investigation on the ICT dynamics of AAQ in an ethanol solution [24]. The TICT dynamics of AAQ of 75–240 fs appear excitation-dependent while the transient absorption measurements, with much less excess vibrational energy, resolved the PICT dynamics of 4.1 ps. The TICT state appears to decay to the ground state and the emitting PICT state decays to the triplet state, via intersystem crossing. In this work, an ultrafast TICT dynamics (110 fs) of AAQ in DMSO has similarly been identified using the FSRS measurements in addition to the vibrational relaxation dynamics of 3.1 ps and a population decay of ~200 ps for the TICT state. The detailed dynamics of AAQ in the TICT state were first measured using a time-resolved vibrational probe of FSRS where AAQ with NH_2_-twisted geometry appears highly sensitive. The ESA and SE bands of AAQ in transient absorption demonstrate biexponential growth, wherein both the ultrafast PICT dynamics of 150–180 fs and the vibrational relaxation dynamics in the PICT state (3.7 ps) were observed in addition to the population decay (560 ps) to the ground state. Detailed ICT dynamics of the AAQ between the TICT and PICT have not been successfully separable through our transient absorption measurements with the 403 and 500 nm excitations. Further exploration by FSRS using the relaxed excitation of 500 nm may resolve the vibrational features of AAQ in the PICT state.

## 3. Materials and Methods

### 3.1. General

The AAQ and 2AAQ from Tokyo Chemical Industry (Tokyo, Japan), and DMSO from Daejung Pure Chemicals (Siheung, Korea) were all used without further purification. The AAQ and 2AAQ solutions of 20–48 mM in DMSO were used for FSRS measurements and dilute (0.4–1 mM) DMSO solutions for the transient absorption measurements. The samples were recirculated with a peristaltic pump to minimize the photodamage or thermal effects, and a 0.5 mm-thick quartz flow cell was used.

### 3.2. Femtosecond Stimulated Raman Setup 

An FSRS setup based on a 1 kHz Ti:sapphire regenerative amplifier was used, and the details of the setup as described elsewhere [48,56]. The broadband Raman probe was generated via supercontinuum generation in a YAG crystal (4 mm thick, Newlight Photonics, Toronto, Canada) and filtered through a long pass filter (830 DCLP, Omega Optical, Brattleboro, VT, USA). The picosecond narrowband Raman pump (800 nm, 0.5 μJ) was prepared in a home-built grating filter with a 1200 gr/mm grating, and was modulated by an optical chopper (MC2000, Thorlabs Inc., Newton, NJ, USA). The bandwidth of the Raman pump was adjusted to keep the spectral resolution at less than 10 cm^−1^. The actinic pump (403 nm, 0.25 μJ) was produced by second-harmonic generation in a beta barium borate (BBO) crystal (*θ* = 29.2°, *ϕ* = 90°, 0.1 mm thick; Eksma Optics, Vilnius, Lithuania) and compressed by a chirped mirror pair (−25 fs^2^ group delay dispersion, Layertec GmbH, Mellingen, Germany). The Raman probe was measured using a fast CCD detector (PIXIS 100, Princeton Instruments, Trenton, NJ, USA) attached to an *f* = 320 spectrograph (Triax 320, Horiba Jobin Yvon, Tokyo, Japan).

### 3.3. Femtosecond Transient Absorption Setup

A home-built transient absorption setup with a broadband visible probe (430–800 nm) was used to obtain the time-resolved electronic spectra. The details of the transient absorption setup have been described elsewhere [57,71]. The actinic pump at 500 nm with a short pulse-width of ~21 fs was prepared in a home-built non-collinear optical parametric amplifier and a pulse compressor, composed of two pairs of the chirped mirrors (−22 fs^2^ group delay dispersion, Layertec GmbH) and a pair of fused silica dispersion wedge prisms (2.8 degrees, Newport, Irvine, CA, USA). A pulse energy of 100 nJ, for both 403 and 500 nm excitations, was used for the transient absorption measurements and a 2 mm-thick quartz cuvette with a stirring magnet was used to minimize the photodamage from the excitation pulses.

### 3.4. Computational Details

The optimized geometries and Raman spectra of AAQ in the ground and excited states were simulated by the density functional theory (DFT) and time-dependent DFT (TDDFT) method using a software package Gaussian 09 [72]. A B3LYP hybrid exchange correlation functional and a 6-311G(d,p) basis set were used with a polarizable continuum model for the solvent effects.

## 4. Conclusions

The intramolecular charge transfer (ICT) of AAQ in the excited state has been measured by time-resolved electronic and vibrational spectroscopy. The ultrafast (150–180 fs) twisted ICT (TICT) dynamics of AAQ, evidenced in the transient absorption measurements, are further confirmed by the transient Raman observations by FSRS, where the TICT dynamics (110 fs) were resolved from the multiple excited-state vibrational modes of ν_C-N_ + δ_NH2_, ν_C=O_, and δ_NH2_. The twist of the NH_2_ group, proposed for the TICT state of AAQ by TDDFT simulations, has been experimentally confirmed by time-resolved spectral changes in the excited-state Raman spectra of AAQ. Furthermore, a strong anharmonic coupling between the TICT reaction coordinate and the low-frequency vibrations of AAQ such as the out-of-plane deformation modes was observed in the form of coherent oscillation signals from the major vibrational modes of AAQ. Lastly, the TICT dynamics, with apparent structural changes in the NH_2_ group, were further observed from the solvation dynamics of DMSO. The ν_S=O_ bands of DMSO, especially in the 1020 cm^−1^ region for the hydrogen-bonded species, showed an ultrafast (60 fs) decrease in intensity and a slower (1.0 ps) recovery during and after the TICT of AAQ. The solvation of the DMSO solvents, including a hydrogen bonding interaction with AAQ and intermolecular interactions between solvent molecules, appears to be a sensitive measure for the excited-state dynamics of solute molecules, especially when accompanying ultrafast structural changes.

## Supplementary Materials

The following are available online at https://www.mdpi.com/article/10.3390/ijms222111926/s1.
