# Supplementary material for: Intramolecular Charge Transfer of 1-Aminoanthraquinone and Ultrafast Solvation Dynamics of Dimethylsulfoxide
Source: Int J Mol Sci. 2021 Nov 3;22(21):11926. doi: 10.3390/ijms222111926 (PMC8584543; doi:10.3390/ijms222111926)
Supplement: Supplementary file 1 [file ijms-22-11926-s001.zip › ijms-1406073-supplementary.pdf]

[Supplementary Materials]

Intramolecular Charge Transfer of 1-Aminoanthraquinone and Ultrafast Solvation Dynamics of Dimethylsulfoxide

Kooknam Jeon,[†] Myungsam Jen,[†] Sebok Lee,[†] Taehyung Jang, and Yoonsoo Pang*

Department of Chemistry, Gwangju Institute of Science and Technology, 123 Cheomdangwagi-ro, Buk-gu, Gwangju 61005, Republic of Korea

Table of Contents

1. Transient absorption of AAQ 2

2. FSRS of AAQ in DMSO solutions 5

3. DFT and TDDFT simulations of AAQ in the ground and excited states 7

4. Data fit for the FSRS results of AAQ 13

5. Nonlinear responses in the solvent vibrational modes of DMSO 14

6. Molecular structures of 1-aminoanthraquinone (AAQ) and 2-aminoanthraquinone (2AAQ) 15

[†]These authors contributed equally to this work.

[*]Author to whom correspondence should be addressed. E-mail address: ypang@gist.ac.kr.

1. Transient absorption of AAQ

Figure S1 shows the transient absorption spectra and kinetics of AAQ in DMSO obtained with the 500 nm excitation. The transient absorption kinetics for the excited state absorption (560 and 600 nm) and the stimulated emission (660 nm) bands were compared in Figure S1(b). From the global analysis, three kinetic components of 150 fs, 3.6 ps, and 640 ps were obtained. The 150 fs dynamics represents the ICT dynamics of AAQ similarly observed as 180 fs with the 403 nm excitation (see Figure 2). A small difference in the ICT dynamics between the results obtained with the 403 nm and 500 nm excitations may be due to the additional vibrational relaxations with excess energy as reported previously in ethanol solution.[1] The difference in the ICT dynamics between the 403 and 500 nm excitation may originate from a slightly better time resolution for the transient absorption measurements with 500 nm (45 fs) compared to the tim resolution (60 fs) with 403 nm excitation. The transient absorption kinetics of AAQ in DMSO were compared between the 403 nm and 500 nm excitations in Figure 2, where the nonlinear response signals around the time delay of zero were removed by subtracting the solvent transient absorption signals. The existence of a common ultrafast ICT dynamics of 150-180 fs regardless of the excitation energy was shown in Figure S2.

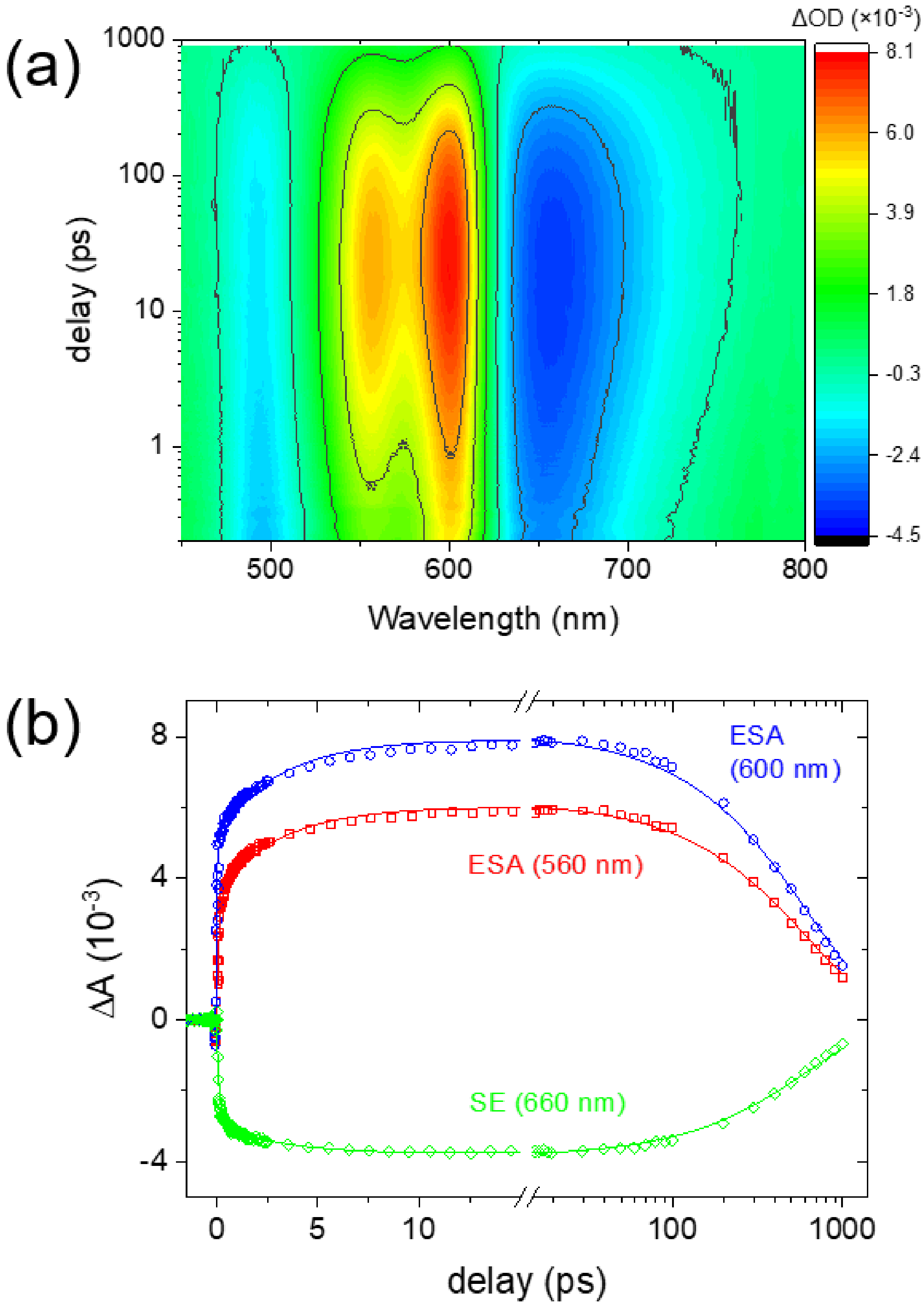

Figure S1. Transient absorption results of 1-aminoanthraquinone (AAQ) in DMSO with 500 nm excitation; (a) surface plot and (b) kinetics for the excited state absorption (ESA) at 560 and 600 nm, and stimulated emission (SE) at 660 nm.

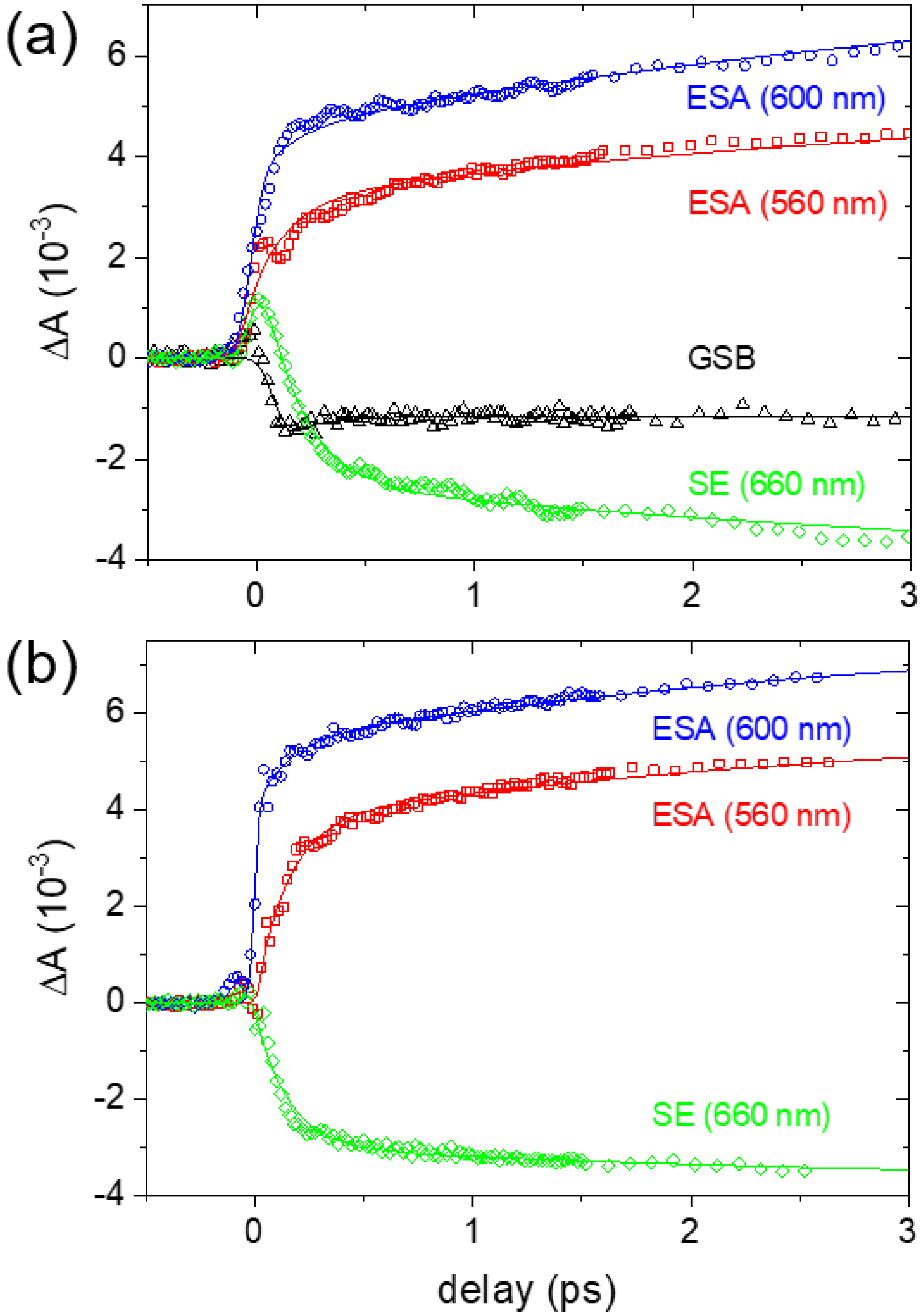

Figure S2. Transient absorption kinetics of AAQ in DMSO solution with (a) 403 nm and (b) 500 nm excitations. The nonlinear response signals around the zero time delay were removed by subtracting the transient absorption kinetics obtained with solvent only.

2. FSRS of AAQ in DMSO solutions

Figure S3 shows the FSRS of AAQ in DMSO solution obtained with the 403 nm excitation. Several excited-state vibrational modes of AAQ appeared in the excited state. The $\delta_{NH2, rocking}$ at 1145 cm^{-1} represents the vibrational mode of AAQ in the locally-excited (LE) S_1 state, many vibrational modes including the δ_{CH} at 1162, $\nu_{C\text{-}N} + \delta_{CH}$ at 1236, ν_{ring} at 1308, and $\nu_{C=O}$ at 1340 cm^{-1} represent the vibrational modes of AAQ in the intramolecular charge transfer (ICT) state of the S_1. The additional bands at 1527 and 1590 cm^{-1} represent the $\nu_{8a} + \nu_{C=O}$ and $\nu_{8a/8b}$ modes of AAQ in the S_1/ICT state.

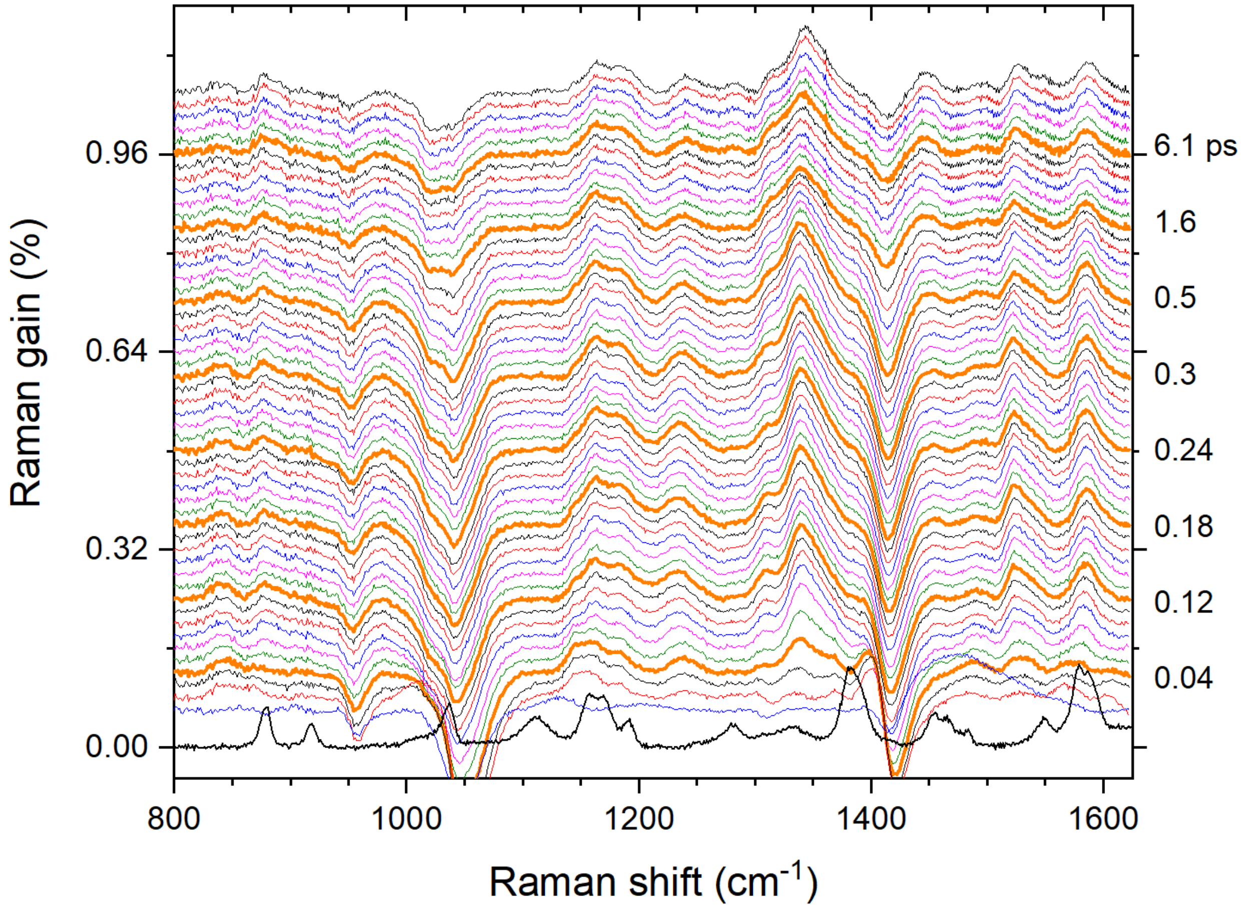

Figure S3. FSRS of AAQ in DMSO solution obtained with 403 nm excitation.

The kinetic traces and peak shifts for the major vibrational modes of AAQ are shown in Figure S4. The ultrafast ICT dynamics of 110 fs is well represented in the $\delta_{NH2,rocking}$ band at 1145 cm^{-1} and the vibrational relaxation dynamics of ~3 ps are overall observed in the major ICT vibrational bands, $\nu_{C\text{-}N} + \delta_{CH}$ at 1236 cm^{-1}, $\nu_{C=O}$ at 1340 cm^{-1}, $\nu_{8a} + \delta_{NH2}$ at 1527 cm^{-1}, and ν_{8b} at 1590 cm^{-1}.

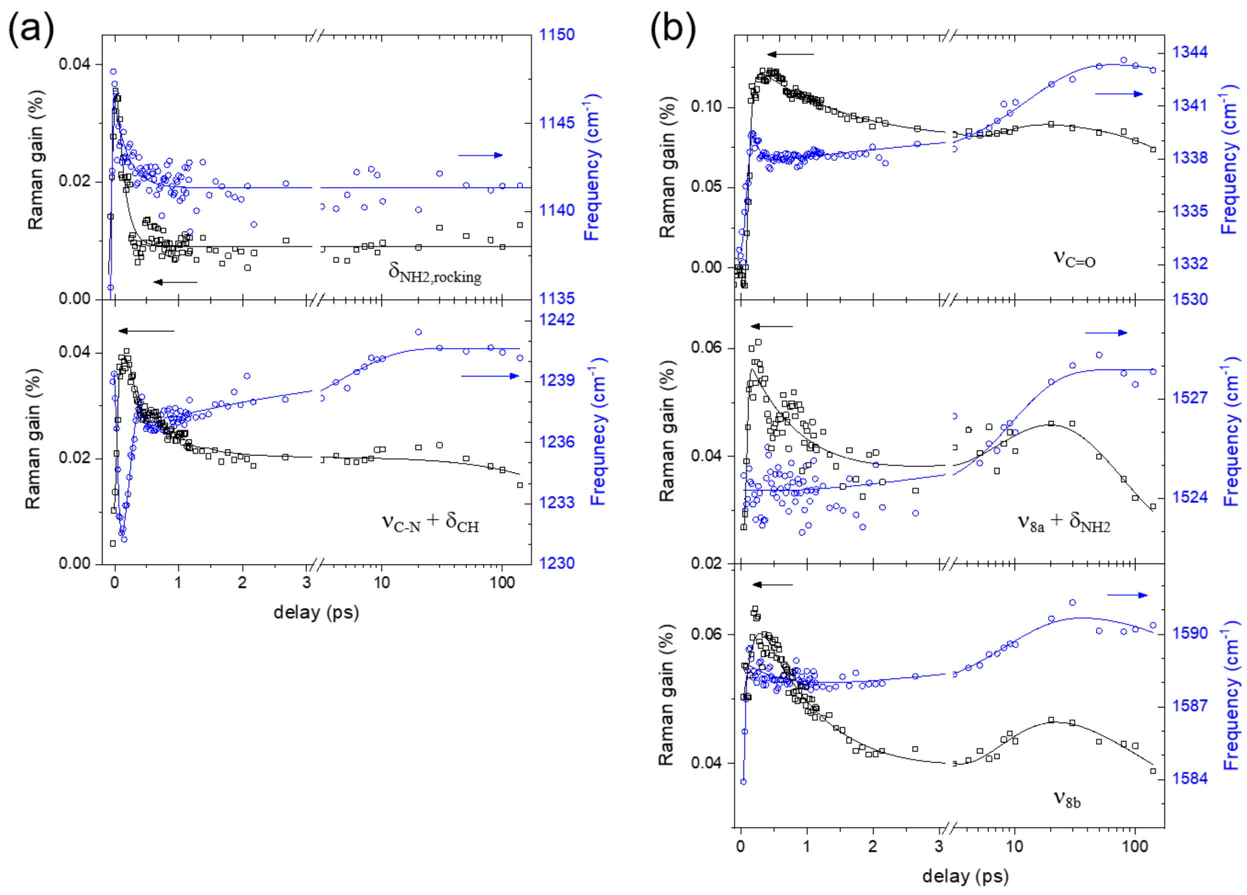

Figure S4. Kinetic traces and structural dynamics of AAQ in DMSO obtained from FSRS measurements with 403 nm excitation; (a) $\delta_{NH2,rocking}$ at 1145 cm^{-1} and $\nu_{C\text{-}N} + \delta_{CH}$ at 1236 cm^{-1}, (b) the major ICT bands of $\nu_{C=O}$ at 1340 cm^{-1}, $\nu_{8a} + \delta_{NH2}$ at 1527 cm^{-1}, and ν_{8b} at 1590 cm^{-1}.

3. DFT and TDDFT simulations of AAQ in the ground and excited states

The electronic structures of AAQ in solution have been investigated by TDDFT simulations and the formation of twisted ICT state with the rotation of amino group has been reported in the S_1 potential surface.[1-3] We performed the optimization of the molecular structure of AAQ in DMSO solution by the DFT and TDDFT simulations at the B3LYP/6-311G(d,p) level with the polarized continuum model (PCM) for DMSO. The optimized structure of AAQ in the ground state appears planar with the planar NH_2 group relative to the anthraquinone backbone as shown in Figure S6(a).

The potential energy surfaces of AAQ in DMSO along the dihedral rotation of NH_2 group were obtained by optimizing the structure of AAQ in the ground state with the several fixed dihedral angles of NH_2 group rotation. Then, the vertical transition energies were calculated by the single point TDDFT simulations at the B3LYP/6-311G(d,p) level with the PCM-DMSO at each optimized geometry with the several specific dihedral angles of NH_2 group rotation. Figure S5 represents the pseudo-potential energy curves of AAQ in DMSO in in the ground state and S_1 excited state. Although the relative energy for the twisted NH_2 geometry appears 0.412 eV higher than the planar NH2 geometry in the S_1 excited state from the pseudo-potential calculations, the existence of the local minimum was found with the twisted NH_2 geometry.

The excited state optimization for the planar or twisted NH_2 group in the S_1 state were done by adopting the initial pseudo geometries of AAQ in the ground state with 0 or 90° NH_2 rotation angle. With the polarized continuum model (PCM) for DMSO, we obtained two optimized structures of AAQ in the S_1 state potential surface as the planar (dihedral angle for NH_2 rotation = 0.0°) and twisted (dihedral angle for NH_2 rotation = 80.7°) conformer as shown in Table S1 and Figure S5. The relative energies of planar and twisted conformers are 2.344 and 2.499 eV,

respectively. Twisted conformer exists higher in energy than the planar conformer by 0.155 eV (1250 cm^{-1}) in DMSO solution. However, the energy ordering between the planar and twisted conformers of AAQ seems to be strongly dependent on the solvent or the simulation methods; twisted conformer was more stable in acetonitrile by 0.105 eV (847 cm^{-1})[3] and the energy ordering appeared inconsistent depending on the levels of the TDDFT method.[1-2]

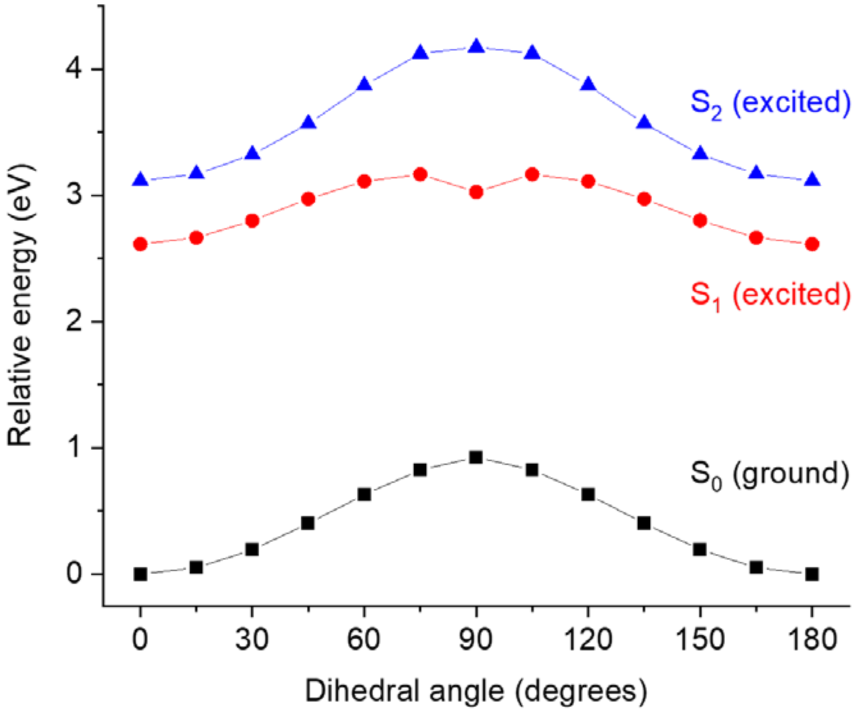

Figure S5. The relative energies at each degree of $-NH_2$ rotation between the planer and twisted structure from ground (S_0) to excited (S_1, S_2) state

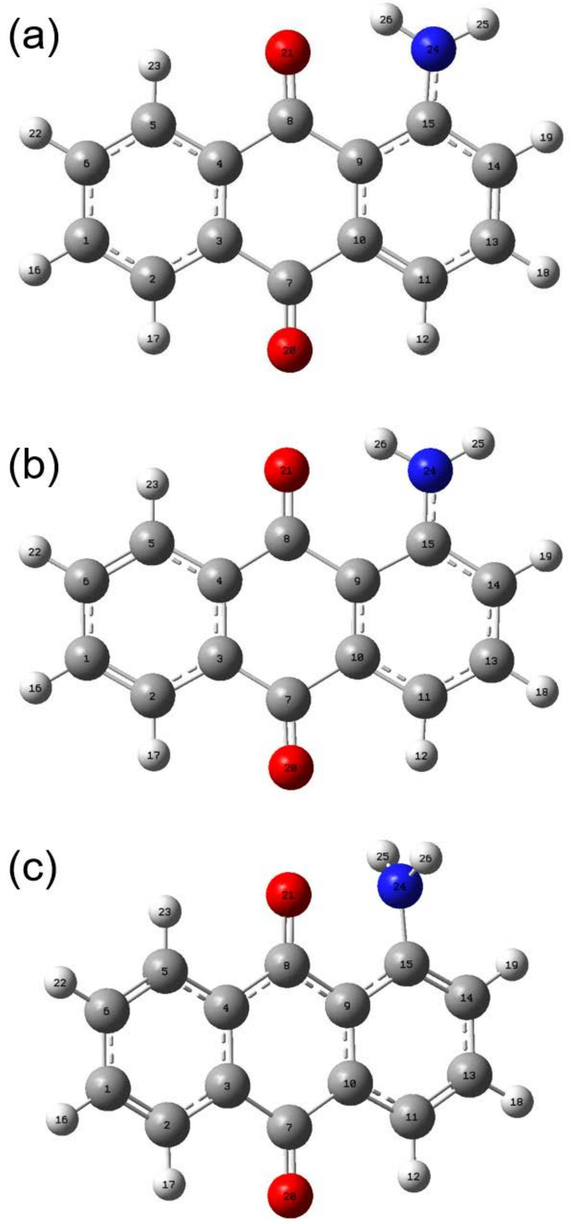

Figure S6. Optimized structures of AAQ in the S_1 excited state by the TDDFT simulations at the B3LYP/6-311G(d,p) level.

Table S1. Optimized geometries of AAQ in the ground (S_0) and the S_1 excited state by the DFT and TDDFT simulations at the B3LYP/6-311G(d,p) level

dihedral angle (degrees)	ground state (S_0)	excited state (S_1)	
		planar	*twisted*
C9-C15-N24-H25	-179.967	-179.997	-99.292
C9-C15-N24-H26	-0.032	-0.008	99.297
C14-C15-N24-H25	0.038	0.003	80.707
C14-C15-N24-H26	179.973	179.993	-80.704
average	0.003	0.005	80.706

Vibrational frequencies and Raman amplitudes of the planar and twisted conformers of AAQ in the S_1 excited state were obtained by the TDDFT simulations at the B3LYP/6-311g(d,p) level with the PCM model for DMSO. The simulated Raman spectrum of the twisted conformer was shown in Figure S7 together with the experimental Raman spectrum of the ICT conformer of AAQ obtained from DMSO solution. The simulated Raman spectra were prepared by adding the Gaussian bands with a bandwidth of 8 cm^{-1} for all the vibrational modes with the specific frequencies and intensities from the DFT simulations. The frequency scaling factor of 0.967 was used for all the DFT simulation results.[4-5] The vibrational bands for the LE (planar) conformer of AAQ in the S_1 state are the $\delta_{NH2, rocking}$ at 1145 cm^{-1} and another shoulder band at ~1215 cm^{-1} shown in Figure 3(a). The vibrational assignments for the $\delta_{NH2, rocking}$ and the shoulder band at ~1215 cm^{-1} mode were based on the DFT simulation results of the planar conformer of AAQ in the S_1 state. All the vibrational bands of the twisted conformer of AAQ designated in Figure S7 were based on the DFT simulation results for the twisted conformer. It is clear that the simulated Raman spectrum of the twisted conformer of AAQ well resembles the experimental Raman spectrum of AAQ in the S_1/ICT state.

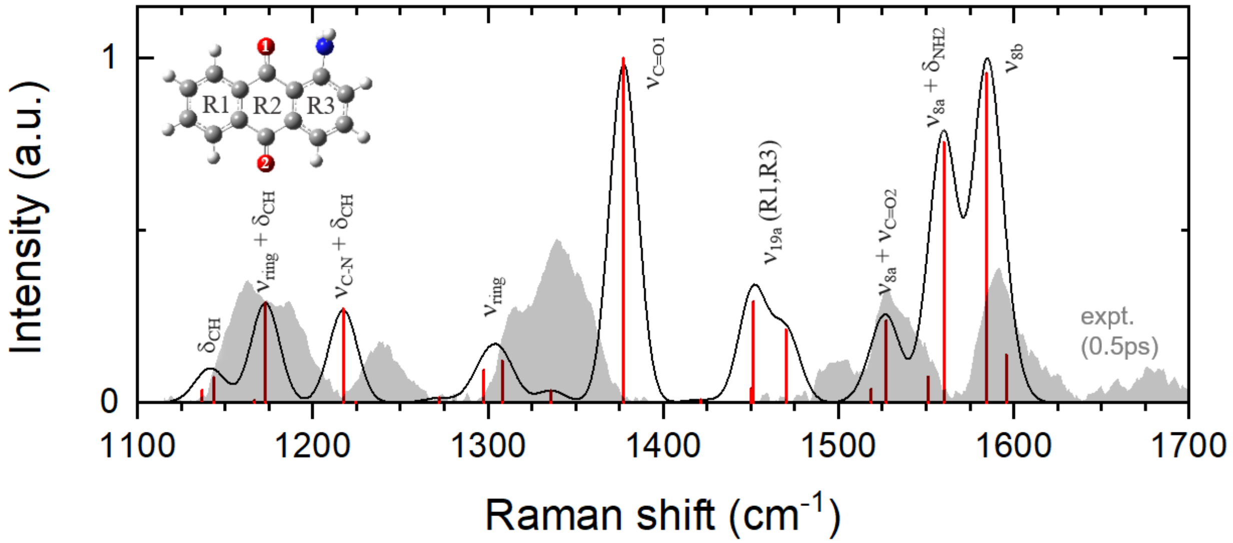

Figure S7. Simulated Raman spectra of AAQ in the twisted ICT state obtained from the TDDFT simulations at the B3LYP/6-311g(d,p) level. Simulated frequencies and amplitudes are marked as red bars and the experimental Raman spectrum of AAQ in the ICT state (at 0.5 ps) was compared.

Figure S8 shows a couple of low frequency vibrational modes of AAQ in the S_1 state with planar geometry of NH_2 group obtained from TDDFT simulations, which visualizes the anharmonic vibrational coupling between these low frequency modes and the transient vibrational modes sensitive to the ICT dynamics of AAQ with the twist of NH_2 group.

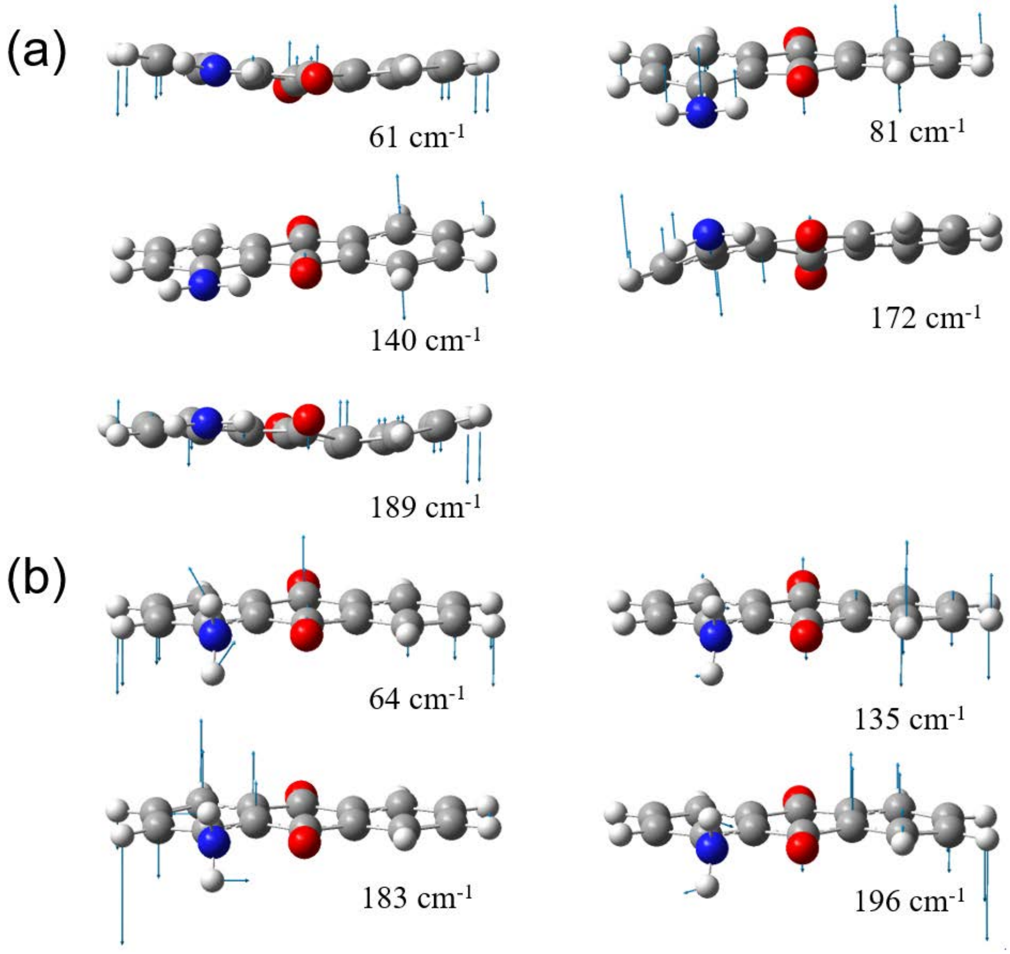

Figure S8. Low frequency vibrational modes of AAQ in the S_1 excited state with (a) the planar and (b) twisted geometry of amino group obtained from the TDDFT simulations at the B3LYP/6-311G(d,p) level with the PCM for DMSO.

4. Data fit for the FSRS results of AAQ

The kinetics of the coherent vibrational signals observed in the FSRS of AAQ in DMSO were fit with the following method. The convoluted exponential-Gaussian function between the Gaussian instrument response function (IRF) and an exponential decay which is multiplied by a Heaviside step function was used for the kinetic model for the exponential growth and decay,

$$\Delta_{\text{Raman Gain}}(t) = A_0 + \sum_i A_i \exp\left(\frac{\omega^2}{2\tau_i^2} - \frac{t-t_0}{\tau_i}\right)\left[1 - erf\left(\frac{\omega^2 - \tau_i(t-t_0)}{\sqrt{2}\omega\tau_i}\right)\right] \quad \text{(S1)}$$

where t_0 is the position of time zero, and ω is the standard deviation of Gaussian function (FWHM = 2.305 · ω) and τ_i is the lifetime of i-th component. Besides, the coherent oscillation signals were included in the exponential-Gaussian convolution fit as several sinusoidal functions multiplied with a damping exponential function,

$$\begin{aligned}\Delta_{\text{Raman Gain}}(t) = A_0 &+ \sum_i A_i \exp\left(\frac{\omega^2}{2\tau_i^2} - \frac{t-t_0}{\tau_i}\right)\left[1 - erf\left(\frac{\omega^2 - \tau_i(t-t_0)}{\sqrt{2}\omega\tau_i}\right)\right] \\ &+ \sum_i B_i \exp\left(\frac{\omega^2}{2\tau_{D,i}^2} - \frac{t-t_{osc,0}}{\tau_{D,i}}\right)\left[1 - erf\left(\frac{\omega^2 - \tau_{D,i}(t-t_{osc,0})}{\sqrt{2}\omega\tau_{D,i}}\right)\right]\sin\left(2\pi\frac{t-t_{osc,0}}{\tau_{osc,i}}\right)\end{aligned}$$

(S2)

where $t_{osc,0}$ is the position of time zero for the damped oscillation functions, $\tau_{D,i}$ and $\tau_{osc,i}$ are the time constant for damping and the period of each oscillation component.

5. Nonlinear responses in the solvent vibrational modes of DMSO

Figure S9 shows the cross phase modulation signals of the solvent vibrational modes ν_{CSC} and $\nu_{S=O}$ modes of DMSO obtained with the 403 nm excitation. The kinetic traces for the positive and negative dispersive signals of each vibrational band represent the instrument response function of the FSRS experiments.

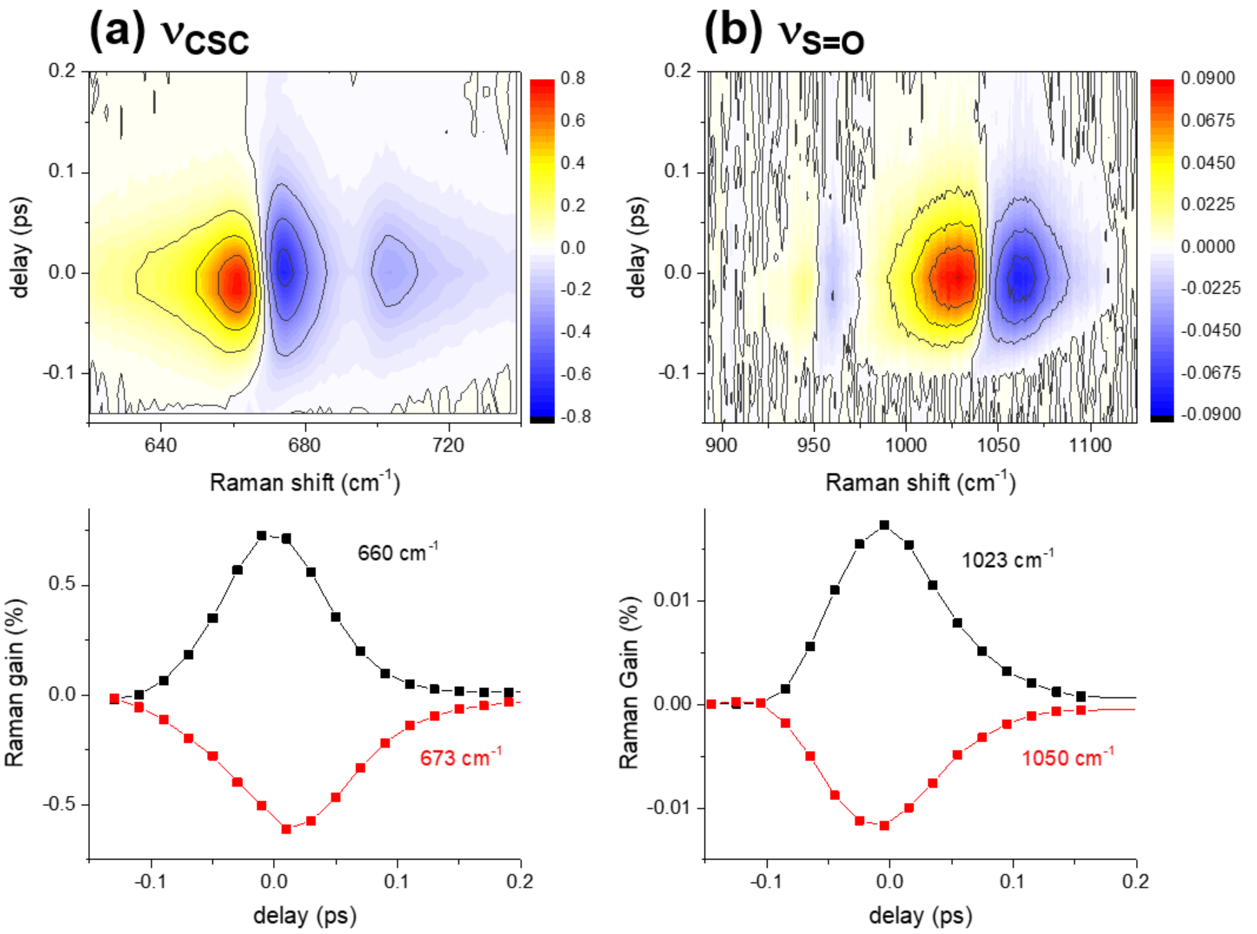

Figure S9. Transient changes in the solvent Raman spectra of DMSO with 403 nm excitation; (a) ν_{CSC} and (b) $\nu_{S=O}$ mode. Kinetic traces for the positive and negative portions of each dispersive cross phase modulation signals are displayed in the lower panels.

6. Molecular structures of 1-aminoanthraquinone (AAQ) and 2-aminoanthraquinone (2AAQ)

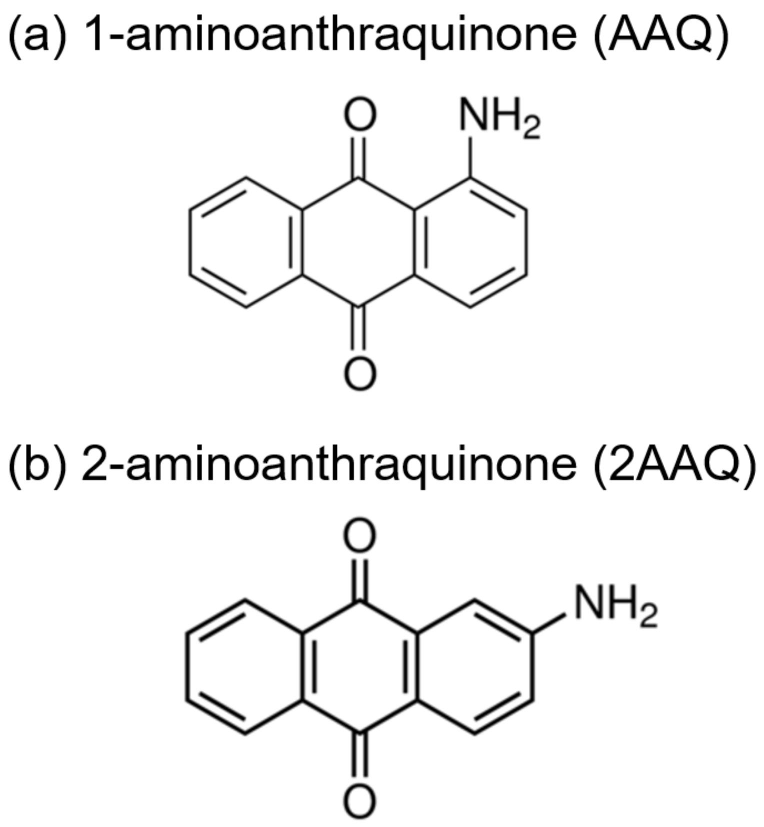

Figure S10. Molecular structures of (a) 1-aminoanthraquinone (AAQ) and (b) 2-aminoanthraquinone (2AAQ)

References

1. Sun, S.; Qin, C.; Liu, H.; Jiang, C., Excitation wavelength dependent ICT character and ISC efficiency in a photocleavage agent of 1-aminoanthraquinone. *Spectrochim. Acta A* **2020,** *234*, 118200.
2. Zhang, S.; Sun, S.; Zhou, M.; Wang, L.; Zhang, B., Ultrafast investigation of photoinduced charge transfer in aminoanthraquinone pharmaceutical product. *Sci. Rep.* **2017,** *7*, 43419.
3. Zhao, Y.; Wang, M.; Zhou, P.; Yang, S.; Liu, Y.; Yang, C.; Yang, Y., Mechanism of Fluorescence Quenching by Acylamino Twist in the Excited State for 1-(Acylamino)anthraquinones. *J. Phys. Chem. A* **2018,** *122* (11), 2864-2870.
4. Irikura, K. K.; Johnson Iii, R. D.; Kacker, R. N., Uncertainties in scaling factors for ab initio vibrational frequencies. *J. Phys. Chem. A* **2005,** *109* (37), 8430-8437.
5. Johnson III, R. D., *NIST Computational Chemistry Comparison and Benchmark Database, NIST Standard Reference Database Number 101, Release 21* **1999**.
